# Lung Tumor Microenvironment Induces Specific Gene Expression Signature in Intratumoral NK Cells

**DOI:** 10.3389/fimmu.2013.00019

**Published:** 2013-02-04

**Authors:** Mélanie Gillard-Bocquet, Charles Caer, Nicolas Cagnard, Lucile Crozet, Mikael Perez, Wolf Herman Fridman, Catherine Sautès-Fridman, Isabelle Cremer

**Affiliations:** ^1^Centre de Recherche des Cordeliers, INSERM, U872Paris, France; ^2^Université Pierre et Marie CurieParis, France; ^3^Université Paris DescartesParis, France; ^4^Plate-Forme de Bioinformatique, Université Paris DescartesParis, France; ^5^INSERM/IRNEM-IFR94Paris, France

**Keywords:** NK cells, lung cancer, microarray

## Abstract

Natural killer (NK) cells are able to recognize and kill tumor cells, however whether they contribute to tumor immunosurveillance is still debated. Our previous studies demonstrated the presence of NK cells in human lung tumors. Their comparison with NK cells from non-tumoral lung tissues and with blood NK cells from the same individuals revealed a decreased expression of some NK receptors and impaired *ex vivo* cytotoxic functions occurring specifically in NK cells isolated from the tumor microenvironment. The aim of the present study was to characterize the transcriptional profile of such intratumoral NK cells, by comparative microarray analysis of sorted NK cells isolated from non-tumoral (Non-Tum-NK) and tumoral (Tum-NK) lung tissues of 12 Non-Small Cell Lung Cancer patients. Our results reveal a specific gene expression signature of Tum-NK cells particularly in activation processes and cytotoxicity, confirming that tumor environment induces modifications in NK cells biology. Indeed, intratumoral NK cells display higher expression levels of NKp44, NKG2A, Granzymes A and K, and Fas mRNA. A particular pattern of receptors involved in chemotaxis was also observed, with an overexpression of CXCR5 and CXCR6, and a lower expression of CX3CR1 and S1PR1 genes in Tum-NK as compared to Non-Tum-NK cells. The precise identification of the molecular pathways modulated in the tumor environment will help to decipher the role of NK cells in tumor immunosurveillance and will open future investigations to manipulate their antitumoral functions.

## Introduction

Among the actors of antitumor immunity, Natural Killer (NK) cells are innate immune cells specialized in recognition and killing of tumor cells, since they have the ability to distinguish normal cells from tumor cells (Vivier et al., 2012). This tumor cell recognition process by NK cells is controlled by an array of activating and inhibitory receptors, which enable NK cells to detect and then kill their cellular targets.

Several experimental evidences demonstrated the important role of NK cells in the elimination of tumor cells. A low NK cell cytotoxicity in peripheral blood was correlated with an increased cancer risk (Vivier et al., 2012). In addition, the presence of tumor infiltrating NK cells, determined as CD57^+^ cells, was associated with good prognosis in colorectal (Coca et al., 1997), gastric (Ishigami et al., 2000), and lung (Villegas et al., 2002) cancers. However, recent studies conducted on NK cells from lung, breast cancer, melanoma, and gastrointestinal stromal tumors indicated that their phenotype and functions were largely altered, resulting in an impaired ability of NK cells to kill tumoral cells *ex vivo* (Delahaye et al., 2011; Mamessier et al., 2011; Platonova et al., 2011; Pietra et al., 2012).

We previously observed that NK cells are enriched in the tumor microenvironment and mainly localized in the tumor stroma of early stages Non-Small Cell Lung Carcinoma (NSCLC; Platonova et al., 2011). Phenotypic and functional analysis of these intratumoral NK cells showed a decreased expression of NK cell receptors, including NKp30, NKp80, DNAM-1, CD16 and ILT-2, and impaired capacities of degranulation. Interestingly, these altered phenotype and functions were specifically observed in NK cells isolated from the tumor, but neither in NK cells from distant lung tissue or blood from the same patient, and nor in NK cells isolated from other lung pathologies, such as emphysema and bronchial dilatation (Platonova et al., 2011). These results emphasize that the tumor microenvironment induces biological modifications of NK cells, that could be related to different mechanisms, such as down-regulation of NK receptors after target cell recognition and interaction, or their cleavage by metalloproteases released in the tumor microenvironment. Another hypothesis is that NK cells, after being recruited in the tumoral site, could sustain a particular program of differentiation, leading to a distinct phenotype.

Here, we therefore characterized intratumoral NK cells at the mRNA level, and compared the gene expression profile of NK cells sorted from the tumors to that of NK cells sorted from the non-tumoral lung of 12 patients, by microarray analysis. This is the first study investigating gene expression of human NK cells isolated from a solid tumor tissue and its non-tumoral counterpart, thus allowing the characterization of NK cells inside their tumor microenvironment. We demonstrate that, among the 42,405 probes representing the whole genome, 1236 genes are differentially expressed, with 792 genes up-regulated, and 444 down-regulated in intratumoral NK cells, indicating a specific transcriptional signature induced by the tumor environment.

## Results

### NSCLC patients and NK cell sorting

The 12 NSCLC patients (stages IB–IIIA) enrolled in this study were smokers and did not receive neo-adjuvant chemotherapy. Among these patients, seven had adenocarcinoma (ADC) and five squamous cell carcinoma (SCC; Table 1).

**Table 1:** **Clinical characteristics of NSCLC patients**.

Patient	Age	Sex	Tobacco (PY)	Histology	Stage (2009)
P1	61	M	45	ADC	IB
P2	62	F	50	ADC	IB
P3	59	F	>10	ADC	IB
P4	72	F	60	ADC	IB
P5	78	F	50	ADC	IB
P6	60	M	50	ADC	IIA
P7	61	M	50	ADC	IIIA
P8	71	F	40	SCC	IB
P9	57	M	80	SCC	IB
P10	79	M	50	SCC	IIB
P11	80	M	40	SCC	IIB
P12	84	M	>10	SCC	IIIA

*Pathological staging and histological types of the tumors were determined according to the 2009 TNM staging system and World Health Organization. PY, packs per year; ADC, adenocarcinoma; SCC, squamous cell carcinoma*.

**Figure 1:**
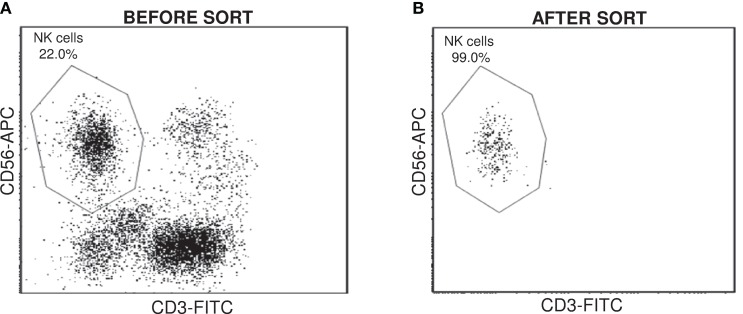
**Cell sorting of NK cells from NSCLC tissues**. CD3^−^CD56^+^ NK cells were sorted from non-tumoral and tumoral tissues for each patient. Cell surface expression was monitored by flow cytometry before **(A)** and after the cell sorting **(B)** to check the purity.

For each patient, NK cell were sorted from tumoral and non-tumoral distant tissue, using the specific immunostaining CD3^−^CD56^+^ (Figure 1). Sorted populations containing more than 95% CD3^−^CD56^+^ cells were considered acceptable to include the sample in the study. Total RNA from Non-Tum-NK and Tum-NK cells was used to analyze whole genome expression by microarray experiments.

### Genome-wide expression comparison between intratumoral and non-tumoral NK cells

Previous studies showed that NK cell phenotype and functions were altered in human lung tumors (Platonova et al., 2011). Gene expression profiling using Agilent microarray technology was performed to characterize the transcriptional signature of Tum-NK and Non-Tum-NK cells. Normalized signal values corresponding to the 42,405 probe-sets representing the whole genome were imported from microarrays data files. Probes (35,120) giving normalized signal values different from the background signal in at least half of the chips involved in the study were selected.

**Figure 2:**
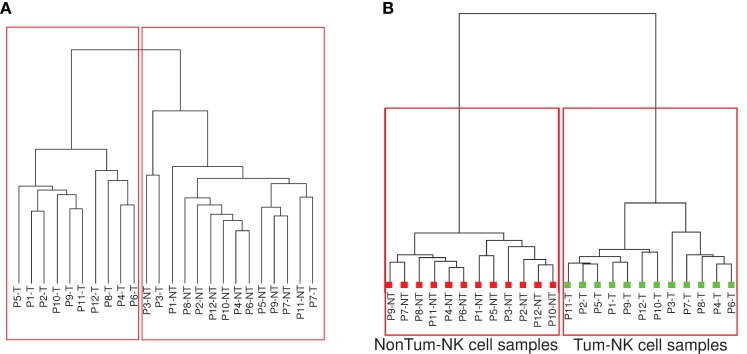
**Clustering analysis on gene expression**. **(A)** After normalization, probes for which fluorescence values were significantly different from the background signals in at least half of the arrays involved in the study were selected (35,120 probes among the total of 42,405 probe-sets). Unsupervised hierarchical clustering based on the 35,120 probes was performed. **(B)** Supervised and unpaired hierarchical clustering was performed based on the 1236 probes that appeared differentially expressed between the Tum-NK and the Non-Tum-NK cells using as filter a minimal twofold-change in expression with *p*-value < 0.05.

To determine whether Tum-NK cells displayed a specific transcriptomic signature, a non-supervised clustering analysis (Spearman correlation similarity measure and ward linkage algorithm) with the normalized signal values of the 35,120 probe-sets was performed (Figure 2A). Interestingly, a clear good delineation between the Tum-NK and Non-Tum-NK cell samples was observed, independently of the patient, except for two of them (Patients 3 and 7).

Based on these results, an unpaired supervised clustering was generated including probes showing a minimal twofold-change expression between Tum-NK and Non-Tum-NK cells, with a minimal *p*-value of 0.05. Using these filters, a list of 1236 genes that were differentially and significantly expressed between Tum-NK and Non-Tum-NK cells was obtained (Figure 2B). A perfect delineation between the Tum-NK and Non-Tum-NK cells was observed. However, there was no clustering of the patients based on smoking status, histological, or stage of the tumor, demonstrating that Tum-NK cells have a distinct gene expression signature compared to Non-Tum-NK cells.

**Figure 3:**
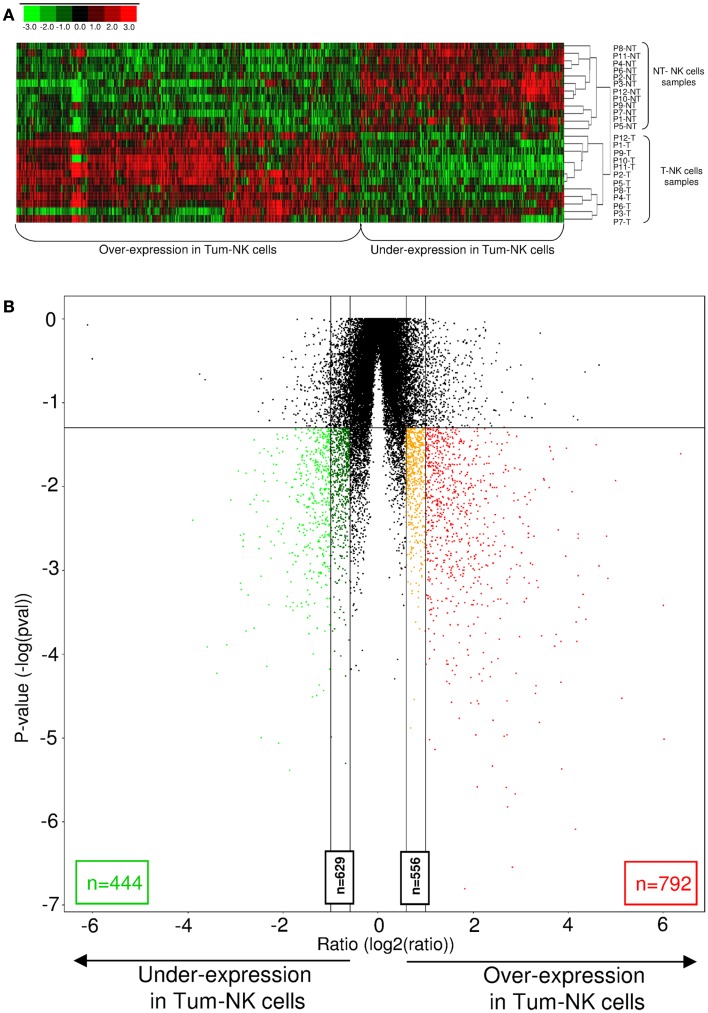
**Differences in genome-wide expression between Tum-NK cells and Non-Tum-NK cells**. **(A)**. Hierarchical clustering of the 1236 differentially expressed genes in Tum-NK cells compared to Non-Tum-NK cells. Individual patients are oriented in rows and expression level for each gene is oriented in columns. Red, green, and black colors of the heat map represent the expression levels that are greater than, equal to, or less than the mean expression levels, respectively, in all samples. **(B)** Volcano plot comparison of differentially expressed genes between Tum-NK cells vs. Non-Tum-NK cells. Genes are arranged along dimensions of biological and statistical significance. The horizontal dimension is the variation of gene expression [log2 (ratio)] between the two groups, and the vertical axis represents the *p*-value. In red and orange are represented genes that are over-expressed in Tum-NK cells, with a fold-change superior to 2 (792 genes) and comprised between 1.5 and 2 (556 genes), respectively. In green and dark green are represented genes that are under-expressed in Tum-NK cells, with a fold-change superior to 2 (444 genes) and comprised between 1.5 and 2 (629 genes), respectively.

To emphasize this observation, a heat map including the 1236 genes differentially expressed between the two groups of NK cells (fold-change >2, *p*-value < 0.05) was built (Figure 3A). All Non-Tum-NK cells samples are clustered in homogenous manner on the upper part of the map, and Tum-NK cells on the down part. A volcano plot showing gene expression variation in Tum-NK cells compared to Non-Tum-NK cells revealed a set of 792 genes that were up-regulated and 444 genes down-regulated in Tum-NK cells (fold-change >2, *p* < 0.05; Figure 3B).

Taken together, these results clearly demonstrated that NK cells sorted from lung tumors display a specific gene expression signature, different from that of NK cells sorted from the non-tumoral lungs.

### Intratumoral NK cells display gene expression signature of activated cells

To determine the immunological meaning of this gene expression profile, the most relevant functions related to genes modulated in Tum-NK were characterized using Ingenuity Pathway Analysis (IPA) application.

Among the 1236 genes, 871 unique and predicted genes were successfully mapped to a gene in Ingenuity Pathways Knowledge Base (IPKB). Networks were then generated from these 871 genes to determine the associated functions including immune cell trafficking and inflammatory responses. The IPA software was used to assort the corresponding list of the top immunological functions based on the number of genes implicated and the *p*-value attributed to the variation of each gene expression (Table 2).

**Table 2:** **Functions modulated in Tum-NK cells**.

Functions	*p*-Value
Cell movement of leukocytes	3.81E−09
Activation of leukocytes	8.53E−06
Cell movement of natural killer cells	3.55E−06
Activation of natural killer cells	4.12E−08
Cytotoxicity of natural killer cells	2.21E−08

The top functions found modulated in Tum-NK cells as compared to Non-Tum-NK cells included mainly movement and activation of leukocytes. Focusing the analysis on NK cell functions, cytotoxicity also appeared as highly significant. Indeed, intratumoral NK cells display a high gene expression of NKp44, a marker that has been demonstrated as an NK cell activation marker (Vitale et al., 1998), according to the overexpression observed by FACS analysis in our previous study, and an increased expression of cytotoxic molecules, such as Granzymes A and K and Fas, compared to NK cells from normal lung tissue. On the contrary, we observed only a slight decreased expression of NKp30, NKp80, DNAM, and CD16 on Tum-NK cells, in contrast with the strong decrease of cell surface expression of these molecules (Platonova et al., 2011). Finally, intratumoral NK cells displayed a specific modulation of gene expression of receptors involved in cell migration, as indicated by the down-regulation of CX3CR1 and S1PR1 and the up-regulation of CXCR5 and CXCR6 receptors (Table 3).

**Table 3:** **Specific genes modulated in Tum-NK cells**.

Genes	Fold-change expression*	*p*-Value
NCR1 (NKp46)	−1.24	3.28E−01
NCR2 (NKp44)	+28.65	7.91E−04
NCR3 (NKp30)	−1.12	8.53E−06
KLRF-1 (NKp80)	−1.8	8.44E−03
DNAM-1 (CD226)	−1.68	8.47E−04
CD32-FCGR2A	−1.4	7.36E−02
CD16-FCGR3A	−1.8	2.68E−03
NKG2A	+2.65	3.95E−03
GZMA	+2.35	3.28E−03
GZMK	+3.31	1.81E−02
Fas	+2.40	1.86E−02
CX3CR1	−2.69	3.65E−03
CXCR5	+4.06	7.08E−03
CXCR6	+3.79	3.97E−03
S1PR1	−2.03	5.96E−03

*Fold-change expression calculated between expression in Tum-NK vs. Non-Tum-NK.

Altogether, these data indicate that intratumoral NK cells after being recruited in the tumor site, display markers of activation and cytotoxicity.

## Discussion

This study revealed transcriptional modulations occurring specifically in NK cells present in NSCLC tumor environment, with particular gene expression signature. These observations are in agreement with our previous observations demonstrating that lung tumor microenvironment induces modifications in NK cells biology.

Several hypothesis could explain the phenotypic and functional alterations of Tum-NK cells. It could reflect that NK cells enter in a particular transcriptional program in the tumor environment or that the recognition of tumor target cells induces these changes. The cleavage of the NK receptors by metalloproteases, or their down-regulation by secreted factors such as IDO and cytokines could represent some of these mechanisms (Mamessier et al., 2011).

In the present microarray analysis, comparing gene expression profile of NK cells sorted from tumoral and non-tumoral lung tissues, we observed only a slight modulation of NKp30, NKp80, CD16, DNAM-1, and ILT-2 at the transcriptional level. This suggests that the low expression of these receptors, which was observed by flow cytometry analysis, was not a result of a particular program of NK cell differentiation induced by the tumor. Therefore investigations have now to be performed to characterize the mechanisms leading to modification of phenotype and functions of NK cells. We identified biological functions associated to modulated genes in Tum-NK cells compared to Non-Tum-NK cells, including activation, cytotoxicity, and leukocyte movement. Among genes that were over-expressed in intratumoral NK cells, we found the NK cell activation marker NKp44, Granzymes A and K, and Fas, which are involved in cytotoxicity, and CXCR5 and CXCR6, two chemokine receptors whose expression has been previously described in few studies in some NK cell subpopulations, such as memory (Paust et al., 2010) or activated NK cells (Berahovich et al., 2006; Dybkaer et al., 2007).

Therefore, this study strongly suggests that NK cells after being recruited in the tumor, become activated and finally exhausted, after target cell recognition. We hypothesize that NK cells may downregulate some receptors at the surface level but not at the transcriptional level as a consequence of such activation, by a mechanism dependent of the tumor environment (Platonova et al., 2011; Cremer et al., 2012). Further investigations will help to define more precisely the genes involved in such functions at the molecular as well as functional levels. Indeed, the precise identification of the molecular functions and pathways modulated in tumor environment will help to decipher the role of NK cells in the control of lung tumors and will open future investigations to manipulate their antitumoral functions.

## Materials and Methods

### Tissue samples from NSCLC patients

Human primary NSCLC samples and non-tumoral distant tissues (situated at >10 cm from the tumor) were obtained from non-treated patients, the day of surgery, at Institut Mutualiste Montsouris (Paris) or Hotel Dieu hospital (Paris). All the patients gave an informed consent. The study was conducted with the agreement of the french ethic comity (number 2008-133) in application with the article L. 1121-1 of french law.

### Preparation of human single-cell suspension and NK cell sorting

Surgical samples were mechanically dilacerated, and single-cell suspensions obtained after enzymatic disruption using collagenase and DNAse for 1 h at 37°C under magnetic agitation in serum-free RPMI1640 RPMI +1% penicillin-streptomycin were filtered through a 0.7 μm cell strainer (BD Biosciences). Cells were washed in PBS + 5% FCS + EDTA 0.5 mM and mononuclear cells were purified using Ficoll gradient.

Mononuclear cells were incubated with fluorescein isothiocyanate – conjugated anti-CD3 (mIgG1κ, clone UCHT1, BD Pharmingen), phycoerythrin-conjugated anti-CD45 (mIgG1κ, clone J.33, Beckman Coulter), and allophycocyanin – conjugated anti-CD56 (mIgG2bκ, clone N-CAM16.2, BD Pharmingen) for 20 min at 4°C and analyzed with a FACSAria III cell sorter (BD Biosciences).

Flow cytometry data were analyzed using Cellquest Pro software (BD Biosciences). NK cells were defined as CD3^−^CD56^+^ cells and sorted in the lysis buffer supplied in the RNA extraction Kit. The purity for the sorted samples was at least 95%. Depending on samples, the number of sorted NK cells ranged between 11,500 and 84,500.

### Isolation of total RNA from NK cells

Total RNA was extracted with the RNEasy Micro Kit (Qiagen) according to manufacturer’s instructions. RNA quality and quantity were analyzed on a PicoChip (Total Eukaryote RNA Assay Pico II Kit, Qiagen) by capillary electrophoresis (BioAnalyzer; Agilent). Samples with a RNA Integrity Number (RIN) ≥7 were considered suitable for microarray experiments.

### Microarray experiments

For the generation of amplified cDNA (SuperAmp Service, Miltenyi Biotec), the mRNA was extracted from the total RNA samples (5 ng) using magnetic beads and transcribed into cDNA using tagged random and oligo(dT) primer. First strand cDNA was 5′ tagged using 8 U terminal deoxynucleotidyl transferase (Fermentas) and incubating for 60 min at 37°C before heat inactivating at 70°C for 5 min. Tagged cDNA was globally amplified (Expand Long Template PCR System DNA Pol Mix, Roche) using primer complementary to the tag sequence and incubating at 78°C for 30 s, 20 cycles of 94°C for 15 s, 65°C for 30 s, and 68°C for 2 min followed by 21 cycles of 94°C for 15 s, 65°C for 30 s, and 68°C for 2.5 min with an extension of 10 s/cycle and a final step of 68°C for 10 min. PCR product was purified (NucleoSpin^®^ Extract II, Macherey-Nagel) and cDNA yield measured. About 250 ng of purified PCR product was labeled with Cy3-dCTP (Amersham) in a Klenow Fragment (10 U) reaction for 1:50 h at 37°C before inactivating through addition of 5 μl 0.5 M EDTA pH 8.0. About 0.5 μg Cy3-labeled and purified (Cy-Scribe GFX Purification Kit, GE Healthcare) cDNAs were used for each microarray analysis.

### Hybridization of agilent whole human genome oligo microarrays

The hybridization procedure was performed according to the One-Color Microarray-Based Gene Expression Analysis protocol (version 6.5, part number G4140-90040) using the Agilent Gene Expression Hybridization Kit (Agilent Technologies). Briefly, 0.5 μg Cy3-labeled cDNA in hybridization buffer was hybridized overnight (17 h, 65°C) to Agilent Whole Human Genome Oligo Microarrays 8 K × 60 K using Agilent’s recommended hybridization chamber and oven. Following hybridization, the microarrays were washed once with the Agilent Gene Expression Wash Buffer 1 for 1 min at room temperature followed by a second wash with preheated Agilent Gene Expression Wash Buffer 2 (37°C) for 1 min. The last washing step was performed with acetonitrile.

### Scanning and data analysis

Fluorescence signals of the hybridized Agilent Microarrays were detected using Agilent’s Microarray Scanner System (Agilent Technologies). The Agilent Feature Extraction Software (FES) 10.7.3.1 was used to read out and process the microarray image files. For determination of differential gene expression FES derived output data files were further analyzed using the Rosetta Resolver gene expression data analysis system (Rosetta Biosoftware, Rosetta error model; Weng et al., 2006).

### Statistical and data analysis

For each probe, the ratio of the average of fluorescence intensity (fold-change) was calculated for the Tum-NK related to the Non-Tum-NK samples.

A “P50” list has been created by filtering probes flagged as “Present” for at least half of the chips involved in the study, and was used for the genome comparison. The group comparisons were done using Student’s *t*-test. To estimate the false discovery rate the resulting *p*-values we filtered at 0.05. Cluster analysis was performed by hierarchical clustering, using the Spearman correlation similarity measure and ward linkage algorithm.

An unsupervised analysis step was performed using clustering prior to any supervised statistical comparison to unveil natural groups among the tested samples.

Probes lists were filtered by *p*-value < 0.05 reflecting the statistical validity and by twofold-change variation of the gene expression. A new clustering step was performed after supervised analysis in order to test the generated list by classifying the samples in expected groups.

To visualize the gene expression we turn mean centered data into heatmaps built by hierarchical classification using the Spearman correlation similarity measure and ward linkage in clustering algorithm. The data were computed with R and the graphic produced with TreeView. A volcano plot was constructed for each comparison results by plotting the negative log of the *p*-value on the *y*-axis and the *x*-axis is the log of the fold-change between the two conditions.

Data were subsequently used for Ingenuity Pathway Analysis (IPA) to model relationships among genes and proteins and to determine putative relevant biological processes.

## Conflict of Interest Statement

The authors declare that the research was conducted in the absence of any commercial or financial relationships that could be construed as a potential conflict of interest.
